# Probabilistic models of genetic variation in structured populations applied to global human studies

**DOI:** 10.1093/bioinformatics/btv641

**Published:** 2015-11-06

**Authors:** Wei Hao, Minsun Song, John D. Storey

**Affiliations:** ^1^ Lewis-Sigler Institute for Integrative Genomics and; ^2^ Center for Statistics and Machine Learning, Princeton University, Princeton, NJ 08544, USA

## Abstract

**Motivation:** Modern population genetics studies typically involve genome-wide genotyping of individuals from a diverse network of ancestries. An important problem is how to formulate and estimate probabilistic models of observed genotypes that account for complex population structure. The most prominent work on this problem has focused on estimating a model of admixture proportions of ancestral populations for each individual. Here, we instead focus on modeling variation of the genotypes without requiring a higher-level admixture interpretation.

**Results:** We formulate two general probabilistic models, and we propose computationally efficient algorithms to estimate them. First, we show how principal component analysis can be utilized to estimate a general model that includes the well-known Pritchard–Stephens–Donnelly admixture model as a special case. Noting some drawbacks of this approach, we introduce a new ‘logistic factor analysis’ framework that seeks to directly model the logit transformation of probabilities underlying observed genotypes in terms of latent variables that capture population structure. We demonstrate these advances on data from the Human Genome Diversity Panel and 1000 Genomes Project, where we are able to identify SNPs that are highly differentiated with respect to structure while making minimal modeling assumptions.

**Availability and Implementation:** A Bioconductor R package called lfa is available at http://www.bioconductor.org/packages/release/bioc/html/lfa.html.

**Contact:** jstorey@princeton.edu

**Supplementary information:** Supplementary data are available at *Bioinformatics* online.

## 1 Introduction

One of the most important goals of modern human genetics is to accurately model genome-wide genetic variation among individuals, as it plays a fundamental role in disease gene mapping and characterizing the evolutionary history of human populations. In this article, we develop latent variable probabilistic models and estimation methods of genetic variation that provide allele frequency estimates of each individual/SNP combination in the presence of arbitrarily complex population structure. Accurate estimates of allele frequencies in this setting allow for improved tests of genetic associations with complex traits and other population genetic analyses which do not rely on overly restricted models of population structure. For example, the models and methods developed here provide the key estimation step in the implementation of a new framework for association testing in the presence of arbitrarily complex structure (Song *etal.*, 2015). Other applications we explore here are to identify loci differentiated with respect to structure, test for random mating in the presence of structure, generalize the estimation of ${\text{F}}_{\text{ST}}$, and characterize the global distribution of allele frequencies of disease SNPs—all making minimal assumptions about the complexity and form of structure.

A longstanding problem has been to provide well-estimated probabilistic models of observed genotypes in the presence of complex population structure (see Raj *etal.*, 2014 and references therein). A series of influential publications have proposed methods to estimate a model of admixture, where the primary focus is on the admixture proportions themselves (Alexander *et al.*, 2009; Pritchard *etal.*, 2000; Tang *etal.*, 2005), which in turn may produce estimates of the allele frequencies of every genetic marker for each individual. Here, we significantly relax the assumptions made about the manifestation of structure to yield more general latent variable models of structure. Rather than targeting admixture proportions, we instead focus on the estimation of the *individual-specific allele frequencies*, and we show that we make significant gains over existing methods in the accuracy and computational efficiency in estimating these quantities. The individual-specific allele frequencies, rather than admixture proportions, are ultimately the key quantities that need to be estimated in the applications we discuss as well as in the association testing method of Song *etal.* (2015).

We propose two flexible genome-wide models of individual-specific allele frequencies as well as methods to estimate them. First, we develop a model that includes as special cases the aforementioned models; specifically, the Balding–Nichols (BN) model (Balding and Nichols, 1995) and its extension to the Pritchard–Stephens–Donnelly (PSD) model (Pritchard *etal.*, 2000). However, we identify some limitations of our method to estimate this model. We therefore propose a second model based on the log-likelihood of the data that allows for rapid estimation of allele frequencies while maintaining a valid probabilistic model of genotypes.

The estimate of the first model is based on principal component analysis (PCA), which is a tool often applied to genome-wide data of genetic variation in order to uncover structure. One of the earliest applications of PCA to population genetic data was carried out by Menozzi *et al.* (1978). Exploratory analysis of complex population structure with PCA has been thoroughly studied (Manni, 2010; Menozzi *etal.*, 1978; Novembre and Stephens, 2008; Rendine *et al.*, 1999; Sokal *et al.*, 1999). We show that a particular application of PCA can also be used to estimate allele frequencies in highly structured populations, although we have to deal with the fact that PCA is a real-valued operation and is not guaranteed to produce allele frequency estimates that lie in the unit interval [0,1].

The estimate of the second model is based on generalized factor analysis approaches that directly model latent structure in observed data, including categorical data (Bartholomew *etal.*, 2011) in which genotypes are included. We utilize a factor model of population structure (Engelhardt and Stephens, 2010) in terms of non-parametric latent variables, and we propose a method called ‘logistic factor analysis’ (LFA) that extends the PCA perspective toward likelihood-based probabilistic models and statistical inference (Collins *etal.*, 2002). LFA is shown to provide accurate and interpretable estimates of individual-specific allele frequencies for a wide range of population structures. At the same time, this proposed approach provides visualizations and numerical summaries of structure similar to that of PCA, building a convenient bridge from exploratory data analysis to probabilistic modeling. LFA plays a key role in the aforementioned new test of genome-wide association of Song *etal.* (2015), called the genotype-conditional association test.

We compare our proposed methods with existing algorithms, ADMIXTURE (Alexander *etal.*, 2009) and fastSTRUCTURE (Raj *etal.*, 2014), and show that when the goal is to estimate all individual-specific allele frequencies, our proposed approaches are conclusively superior in both accuracy and computational speed. We apply the proposed methods to the Human Genome Diversity Project (HGDP) (Cann *etal.*, 2002; Rosenberg *etal.*, 2002, 2005) and 1000 Genomes Project (TGP) (1000 Genomes Project Consortium, 2010) datasets, which allows us to estimate allele frequencies of every SNP in an individual-specific manner. Using LFA, we are also able to rank SNPs for differentiation according to population structure based on the likelihoods of the fitted models. In both datasets, the most differentiated SNP is proximal to *SLC24A5*, and the second most differentiated SNP is proximal to *EDAR.* Variation in both of these genes has been hypothesized to be under positive selection in humans. In the TGP dataset, the second most different SNP is rs3827760, which confers a missense mutation in *EDAR* and has been recently experimentally validated as having a functional role in determining a phenotype (Kamberov *etal.*, 2013). We also identify several SNPs that are highly differentiated in these global human studies that have recently been associated with diseases such as cancer, obesity and asthma.

## 2 Methods

### 2.1 Models of Allele Frequencies

**Fig. 1.**
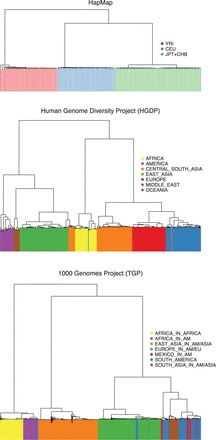
A hierarchical clustering of individuals from the HapMap, HGDP and TGP datasets. A dendrogram was drawn from a hierarchical clustering using Ward distance based on SNP genotypes (MAF >5%). Whereas the HapMap project shows a definitive discrete population structure (by sampling design), the HGDP and TGP data show the complex structure of human populations

It is often the case that human and other outbred populations are ‘structured’ in the sense that the genotype frequencies at a particular locus are not homogeneous throughout the population (Astle and Balding, 2009). Geographic characterizations of ancestry often explain differing genotype frequencies among subpopulations. For example, an individual of European ancestry may receive a particular genotype according to a probability different than an individual of Asian ancestry. This phenomenon has been observed not only across continents, but on very fine scales of geographic characterizations of ancestry. Recent studies have shown that population structure in human populations is quite complex, occurring more on a continuous rather than a discrete basis (Rosenberg *etal.*, 2002). We can illustrate the spectrum of structural complexity with Figure 1, which shows dendrograms of hierarchically clustered individuals from the HapMap (phase II), HGDP and TGP datasets. The HapMap samples strongly indicate explicit membership of each individual to one of three discrete subpopulations (due to the intended sampling scheme). On the other hand, the clusterings of the HGDP and TGP individuals show a very complex configuration, more representative of random sampling of global human populations.

Let us introduce Z as an unobserved variable capturing an individual’s structure, which we will estimate with dimension *d.* Let *x_ij_* be the observed genotype for SNP *i* and individual *j* (i=1,…,m, j=1,…,n), and assume that *x_ij_* is coded to take the values 0, 1, 2. We call the observed *m* × *n* genotype matrix X. For SNP *i*, the allele frequency can be viewed as a function of Z, i.e. $\pi_{i}(Z)$. For a sampled individual *j* from an overall population, we have ‘individual-specific allele frequencies’ (Thornton *etal.*, 2012) defined as $\pi_{ij}\equiv \pi_{i}(z_{j})$ at SNP *i.* Each value of *π_ij_* informs us as to the expectation of that particular SNP/individual pair under the scenario we observed a new individual at that locus with the same structure, specifically as $E[x_{ij}]/2=\pi_{ij}$. If an observed SNP genotype *x_ij_* is treated as a random variable, then we assume that *π_ij_* serves to model *x_ij_* as a Binomial parameter: $x_{ij}|Z=z_{j}∼\text{Binomial}(2,\pi_{i}(z_{j}))$. (We will drop the conditioning on Z in the subsequent text for convenience.) This Binomial distribution assumption is also made in the PSD model (Alexander *etal.*, 2009; Pritchard *etal.*, 2000). The focus of this article is on the simultaneous estimation of the *π_ij_* values (i=1,…,m, j=1,…,n).

The flexible, accurate and computationally efficient estimation of individual-specific allele frequencies is important for population genetic analyses, illustrated by the following examples.

**Example 1**

Corona *et al.* (2013) recently showed that considering the worldwide distribution of allele frequencies of SNPs known to be associated with human diseases may be a fundamental component to understanding the relationship between ancestry and disease.

**Example 2**

We may use individual-specific allele frequency estimates to determine whether genotype data follow a probability distribution indicative of random mating, conditional on population structure. This involves verifying that $x_{ij}|Z=z_{j}∼\text{Binomial}(2,\pi_{ij}(z_{j}))$. Verifying this model can be viewed as testing for a version of Hardy–Weinberg equilibrium conditional on structure; it is also the probabilistic assumption underlying the STRUCTURE (Pritchard *etal.*, 2000), ADMIXTURE and fastSTRUCTURE software packages that all fit the PSD model. Verifying this model assumption can be accomplished by assessing the goodness-of-fit of the model by testing whether the genotype frequencies for SNP *i* follow probabilities $\pi_{ij}^{2},\;2\pi_{ij}(1-\pi_{ij})$, and ${\left(1-\pi_{ij}\right)}^{2}$ for all individuals j=1,…,n.

**Example 3**

It can be shown that an $F_{\text{ST}}$-related measure can be characterized for SNP *i* using values of *π_ij_*, j=1,2,…,n (Supplementary materials, Section S5).

**Example 4**

We have recently developed a test of association that corrects for population structure and involves the estimation of $\log\left(\frac{\pi_{ij}}{1-\pi_{ij}}\right)$ (Song *etal.*, 2015).

These examples demonstrate that flexible and well-behaved estimates of the individual-specific allele frequencies *π_ij_* are needed for downstream population genetic analyses.

It is straightforward to write other models of population structure in terms of Z. For the BN model, each individual is assigned to a population, thus $z_{j}$ indicates individual *j*’s population assignment. For the PSD model, each individual is considered to be an admixture of a finite set of ancestral populations. Following the notation of Pritchard *et al.* (2000), we can write $z_{j}$ as a vector with elements *q_kj_*, where *k* indexes the ancestral populations, and we constrain *q_kj_* to be between 0 and 1 subject to $\sum_{k}q_{kj}=1$. Assuming the PSD model allows us to write each $\pi_{ij}=\sum_{k}p_{ik}q_{kj}$ and leads to a matrix form: F=PQ, where F is the *m* × *n* matrix of allele frequencies with (*i*, *j*) entry *π_ij_*, P is the *m* × *d* matrix of ancestral population allele frequencies *p_ik_* and Q is the *d* × *n* matrix of admixture proportions. The elements of P and Q are explicitly restricted to the range [0,1].

The PSD model is primarily focused on the matrix Q and secondarily on the matrix P, which have standalone interpretations. We aim instead to estimate all *π_ij_* quantities with a high level of accuracy and computational efficiency. Writing the structure of the allele frequency matrix F as a linear basis, we have:

(1) Model 1: F=ΓS,

where Γ is *m* × *d* and S is *d* × *n* with d≤n, and the entries of both matrices are unrestricted real numbers. The *d* × *n* matrix S encapsulates the genetic population structure for these individuals since **S** is not SNP-specific. The *m* × *d* matrix Γ maps how the structure S is manifested in the allele frequencies. Operationally, each SNP’s allele frequencies are a linear combination of the rows of S, where the linear weights for SNP *i* are contained in row *i* of Γ. We define the dimension *d* so that *d* = 1 corresponds to the case of no structure: when *d* = 1, S=(1,1,…,1) and Γ is the column vector of marginal allele frequencies.

This model is not necessarily the most effective way to estimate *π_ij_* when working in the context of a probabilistic model or with the likelihood function given the data. Model 1 resembles linear regression, where the allele frequencies are treated as a real-valued response variable that is linearly dependent on the structure. A version of regression for the case of categorical response variables (e.g. genotypes) with underlying probability parameters is logistic regression. We developed an approach called logistic factor analysis (LFA), which is essentially an extension of non-parametric factor analysis to {0, 1, 2}-valued genotype data. The justification for LFA derives from that of generalized linear models (McCullagh and Nelder, 1989), where in our case observed covariates are instead replaced with unobserved latent variables that must also be estimated.

The log-likelihood is the preferred mathematical framework for representing the information the data contain about unknown parameters (Lehmann and Casella, 1998). Suppose that the model assumption holds such that $x_{ij}∼\text{Binomial}(2,\pi_{ij})$. We can write the log-likelihood of the data for SNP *i* and individual *j* as:

$$\ell (\pi_{ij}|x_{ij})=\log\left(\text{Pr}(x_{ij}|\pi_{ij})\right)\propto \log\left(\pi_{ij}^{x_{ij}}{\left(1-\pi_{ij}\right)}^{2-x_{ij}}\right)=x_{ij}\log\left(\frac{\pi_{ij}}{1-\pi_{ij}}\right)+2\log(1-\pi_{ij}).$$

The log-likelihood of SNP *i* for all unrelated individuals is the sum: $\sum_{j=1}^{n}\ell (\pi_{ij}|x_{ij})$. The term $\log\left(\frac{\pi_{ij}}{1-\pi_{ij}}\right)$ is the logit function and is written as $\text{logit}(\pi_{ij})$. $\text{logit}(\pi_{ij})$ is called the ‘natural parameter’ or ‘canonical parameter’ of the Binomial distribution and is the key component of logistic regression (McCullagh and Nelder, 1989). An immediate benefit of working with $\text{logit}(\pi_{ij})$ is that it is real valued, which allows us to directly model $\text{logit}(\pi_{ij})$ with a linear basis.

Let L be the *m* × *n* matrix with (*i*, *j*) entry equal to $\text{logit}(\pi_{ij})$. We form the following parameterization of L:

(2) Model 2: L=AH,

where A is *m* × *d* and H is *d* × *n* with d≤n. In this case we can write

$$\text{logit}(\pi_{ij})=\sum_{k=1}^{d}a_{ik}h_{kj},$$

where all parameters are free to span the real numbers. We choose the value of *d* by identifying the one that provides the best goodness-of-fit (Supplementary materials, Section S2).

We call the rows of H ‘logistic latent factors’ or just ‘logistic factors’ as they represent unobserved variables that explain the inter-individual differences in allele frequencies. In other words, the logit of the vector of individual-specific allele frequencies for SNP *i* can be written as a linear combination of the rows of H:

$$[\text{logit}(\pi_{i1}),\ldots ,\text{logit}(\pi_{in})]=\text{logit}(\pi_{i})=\sum_{k=1}^{d}a_{ik}h_{k},$$

where $h_{k}$ is the *k*th row of H. Similarly, we can write:

$$(\pi_{i1},\ldots ,\pi_{in})=\pi_{i}=\frac{\exp\left[\sum_{k=1}^{d}a_{ik}h_{k}\right]}{1+\exp\left[\sum_{k=1}^{d}a_{ik}h_{k}\right]}.$$

The relationship between our proposed LFA approach and existing approaches of estimating latent variables in categorical data is detailed in Supplementary materials, Section S6. Specifically, it should be noted that even though we propose calling the approach LFA, we do not make any assumptions about the distribution of the factors (which are often assumed to be normal). A technically more detailed name of the method is a ‘logistic nonparametric linear latent variable model for Binomial data.’

### 2.2 Estimation algorithms

The two models presented earlier make minimal assumptions as to the nature of the structure. For example, in Model 1, both Γ and S are permitted to be real valued. This allows us to apply a PCA-based algorithm directly to the genotype matrix X, obtaining estimates of $\tilde{F},\;\tilde{\Gamma }$ and $\tilde{S}$. In essence, $\tilde{F}$ is estimated by forming the projection of X/2 onto the top *d* principal components of X with an explicit intercept for the *d* = 1 case. One drawback of this approach is that because PCA is designed for continuous data, we have to take additional steps to constrain $\tilde{F}$ to be in the range [0,1]. However, we show in Results that $\tilde{F}$ is still an extremely accurate estimate of the allele frequencies F for all formulations of F considered here, including the PSD model.

**Algorithm 1: Estimating F from PCA**

1. Let ${\tilde{\mu }}_{i}$ be the sample mean of row *i* of X. Set $x_{ij}^{*}=x_{ij}-{\tilde{\mu }}_{i}$ and let $X^{*}$ be the *m* × *n* matrix with (*i*, *j*) entry $x_{ij}^{*}$.
2. Perform singular value decomposition (SVD) on $X^{*}$ which decomposes $X^{*}=U\Delta V^{T}$. Note that the rows of $\Delta V^{\text{T}}$ are the *n* row-wise principal components of $X^{*}$ and U are the principal component loadings.
3. Let ${\tilde{X}}_{d-1}^{*}$ be the projection of $X^{*}$ on the top *d* – 1 eigen-vectors of this SVD, ${\tilde{X}}_{d-1}^{*}=U_{1:(d-1)}\Delta_{1:(d-1)}V_{1:(d-1)}^{T}$.
4. Construct ${\tilde{F}}^{*}$ by adding ${\tilde{\mu }}_{i}$ to row *i* of ${\tilde{X}}_{d-1}^{*}$ (for i=1,…,n) and multiplying the resulting matrix by 1/2. In mathematical terms, ${\tilde{F}}^{*}=\tilde{\Gamma }\tilde{S}$ where
  Γ˜=(12μ˜112U1:(d−1)Δ1:(d−1)⋮12μ˜m)=(12u11δ1⋯12u1,d−1δd−112μ˜112u21δ1⋯12u2,d−1δd−112μ˜2⋮ ⋮⋮12um1δ1⋯12um,d−1δd−112μ˜m),S˜=(V1:(d−1)T1 1 … 1)=(v11v21⋯vn1v12v22⋯vn2⋮⋮ ⋮v1,d−1v2,d−1⋯vn,d−111⋯1),
  and *δ_i_* is the *i*th diagonal entry of Δ. Let ${\tilde{\pi }}_{ij}^{*}$ to be the (*i*, *j*) entry of ${\tilde{F}}^{*}$.
5. Since it may be the case that some ${\tilde{\pi }}_{ij}^{*}$ are such that ${\tilde{\pi }}_{ij}^{*}<0$ or ${\tilde{\pi }}_{ij}^{*}>1$, we truncate these. The final PCA based estimate of F is formed as $\tilde{F}$ where the (*i*, *j*) entry ${\tilde{\pi }}_{ij}$ is defined to be
  $${\tilde{\pi }}_{ij}=\{\begin{array}{ll} C & \text{if}\;{\tilde{\pi }}_{ij}^{*}\leq C\\ {\tilde{\pi }}_{ij}^{*} & \text{if}\;C<{\tilde{\pi }}_{ij}^{*}<1-C\\ 1-C & \text{if}\;{\tilde{\pi }}_{ij}^{*}\geq 1-C \end{array}$$
  for some C≳ 0. An estimate of L can be formed as $\tilde{L}=\text{logit}(\tilde{F})$.

Here we used $C=\frac{1}{2n}$, which is the minimum resolution of the data given 2*n* alleles are observed. In summary, $\tilde{F}$ is a projection of X into its top principal components, scaled by 1/2, and truncated so that all values lie in the interval (0, 1).

For Model 2, we propose a method for estimating the latent variable H. Starting from the output of Algorithm 1, we apply the logit transformation to the subset of rows that had no truncation, i.e. no values where ${\tilde{\pi }}_{ij}^{*}\leq C$ or ${\tilde{\pi }}_{ij}^{*}\geq 1-C$. We then extract the right singular vectors of this transformed subset. As long as the subset is large enough to span the same space as the row space of L, this approach accurately estimates the basis of H. Next, we calculate the maximum likelihood estimation of A parameterized by $\hat{H}$ to yield $\hat{A}$, and then set $\hat{L}=\hat{A}\hat{H}$. This involves performing a logistic regression of each SNP’s data on $\hat{H}$. In order to estimate the individual-specific allele frequency matrix F, we calculate $\hat{F}={\text{logit}}^{-1}(\hat{L})$. An important property to note is that all ${\hat{\pi }}_{ij}\in [0,1]$ due to the fact that we are modeling the natural parameter.

**Algorithm 2: Estimating Logistic Factors**

1. Apply steps 1–4 of Algorithm 1 to obtain the estimate ${\tilde{F}}^{*}$ from Step 4.
2. Recalling that ${\tilde{\pi }}_{ij}^{*}$ is the (*i*, *j*) entry of ${\tilde{F}}^{*}$, we choose some C≳0 and form
  $$S=\{i:C<{\tilde{\pi }}_{ij}^{*}<1-C,\forall j=1,...,n\}.$$
  S identifies the rows of ${\tilde{F}}^{*}$ where the logit function can be applied stably. Here we use $C=\frac{1}{2n}$.
3. Define ${\tilde{F}}_{S}$ to be the corresponding subset of rows of ${\tilde{F}}^{*}$, and calculate ${\tilde{L}}_{S}=\text{logit}\left({\tilde{F}}_{S}\right)$. Let ${\tilde{L}}_{S}\prime$ be the row-wise mean centered and standard deviation scaled matrix ${\tilde{L}}_{S}$.
4. Perform SVD on ${\tilde{L}}_{S}\prime$ resulting in ${\tilde{L}}_{S}\prime =T\Lambda W^{T}$. Set $\hat{H}$ to be the *d* × *n* matrix composed of the top *d* – 1 right singular vectors of the SVD of ${\hat{L}}_{S}\prime$ stacked on the row *n*-vector (1,1,⋯,1):
  H^=( W1:(d−1)T 11⋯11)=(w11w21⋯wn1w12w22⋯wn2⋮⋮ ⋮w1,d−1w2,d−1⋯wn,d−111⋯1).

**Algorithm 3: Estimating F and L from LFA**

1. Apply Algorithm 2 to X to obtain $\hat{H}$.
2. For each SNP *i*, perform a logistic regression of the SNP genotypes $x_{i}=(x_{i1},x_{i2},\ldots ,x_{in})$ on the rows of $\hat{H}$, specifically by maximizing the log-likelihood
  $$\ell (\pi_{i}|x_{i},\hat{H})=\sum_{j=1}^{n}x_{ij}\log\left(\frac{\pi_{ij}}{1-\pi_{ij}}\right)+2\log(1-\pi_{ij})$$
  under the constraint that $\text{logit}(\pi_{ij})=\sum_{k=1}^{d}a_{ik}{\hat{h}}_{kj}$. It should be noted that an intercept is included because ${\hat{h}}_{dj}=1$∀j by construction.
3. Set ${\hat{a}}_{ij}$ (j=1,…,n) to be equal to the maximum likelihood estimates from the above model fit, for each of i=1,…,m. Let $\hat{L}=\hat{A}\hat{H},\;\hat{F}={\text{logit}}^{-1}(\hat{L})$, and ${\hat{\pi }}_{ij}$ be the (*i*, *j*) entry of $\hat{F}$:
  $${\hat{\pi }}_{ij}=\frac{\exp\left\{\sum_{k=1}^{d}{\hat{a}}_{ik}{\hat{h}}_{kj}\right\}}{1+\exp\left\{\sum_{k=1}^{d}{\hat{a}}_{ik}{\hat{h}}_{kj}\right\}}.$$

PCA-based estimation of Model 1 requires one application of SVD and LFA requires two applications of SVD. We leverage the fact that n≫d to utilize Lanczos bidiagonalization which is an iterative method for computing the SVD of a matrix (Baglama and Reichel, 2006). Lanczos bidiagonalization excels at computing a few of the largest singular values and corresponding singular vectors of a sparse matrix. While the sparsity of genotype matrices is fairly low, we find that in practice using this method to perform the above estimation algorithms is more effective than using methods that require the calculation of all the singular values and vectors. This results in a substantial reduction of the computational time needed for the implementation of our methods.

## 3 Results

We applied our methods to a comprehensive set of simulation studies and to the HGDP and TGP datasets.

### 3.1 Simulation studies

To directly evaluate the performance of the estimation methods (see Section 2.2), we devised a simulation study where we generated synthetic genotype data with varying levels of complexity in population structure. Genotypes were simulated based on allele frequencies subject to structure from the BN model, the PSD model, spatially structure populations and real datasets. For the first three types of simulations, the allele frequencies were parameterized by Model 1, while for the real-data simulations, the allele frequencies were taken from model fits on the data themselves.

A key property to assess is how well the estimation methods capture the overall structure. One way to evaluate this is to determine how well $\tilde{S}$ from the PCA-based method (Algorithm 1) estimates the true underlying S, and similarly how well $\hat{H}$ from LFA estimates the true H. Note that even though the genotype data were generated from the F of Model 1, we can evaluate $\hat{H}$ by converting with L=logit(F). To evaluate PCA, we regressed each row of F on $\tilde{S}$ and calculated the average *R*^2^; similarly, for LFA we regressed each row of L on $\hat{H}$ and calculated the average *R*^2^ value. The results are presented in Table 1. Both methods estimate the true latent structure well.

**Table 1.** Accuracy in estimating linear bases for S

Scenario	Mean *R*^2^	
	$F∼\tilde{S}$	$\text{logit}(F)∼\hat{H}$
TGP fit by PCA	0.9998	0.9722
TGP fit by LFA*	0.9912	0.9990
HGDP fit by PCA	0.9996	0.9614
HGDP fit by LFA*	0.9835	0.9983
BN	0.9999	0.9999
PSD α=0.01	0.9998	0.9974
PSD α=0.1	0.9998	0.9879
PSD α=0.5	0.9996	0.9827
PSD *α* = 1	0.9993	0.9844
Spatial *a* = 0.1	0.9999	0.9964
Spatial *a* = 0.25	0.9999	0.9962
Spatial *a* = 0.5	0.9999	0.9964
Spatial *a* = 1	0.9998	0.9970

Column 1 shows the scenario from which the data were simulated. Columns 2 and 3 display the estimation accuracy of the PCA-based method (Column 2) and LFA (Column 3). Column 2 shows the mean *R*^2^ value when regressing the true $\left(\pi_{i1},\pi_{i2},\ldots ,\pi_{in}\right)$ on $\tilde{S}$ from PCA, averaging across all SNPs. Column 3 shows the mean *R*^2^ value when regressing the true $\left(\text{logit}(\pi_{i1}),\text{logit}(\pi_{i2}),\ldots ,\text{logit}(\pi_{in})\right)$ on $\hat{H}$ from LFA, averaging across all SNPs. All estimated standard errors fell between 10^−6^ and 10^−8^ so these are not shown. Note for each scenario, *R*^2^ values are higher for the method from which the true F matrix was generated. All but the two scenarios marked with an asterisk (*) are from Model 1, while the two marked scenarios are from Model 2, where we took $F={\text{logit}}^{-1}L$

**Fig. 2.**
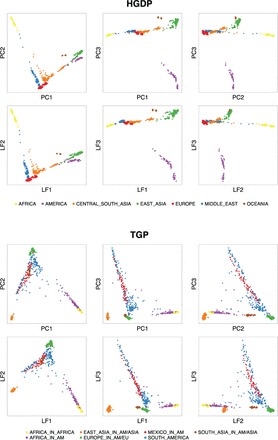
Principal component and logistic factor biplots for the HGDP and TGP datasets. The top three principal components from each dataset are plotted in a pairwise fashion in the top panel. The top three logistic factors are plotted analogously in the bottom panel. It can be seen that both approaches yield similar visualizations of structure

We specifically note that when the PSD model was utilized to simulate structure, we were able to recover the structure S very well (Supplementary Fig. S2) without needing to employ the computationally intensive and assumption-heavy Bayesian model fitting techniques from Pritchard *etal.* (2000). In addition, it seems that the $\tilde{S}$ largely captures the geometry of S where it may be the case that S can be recovered with a high degree of accuracy by transforming $\tilde{S}$ back into the simplex. By comparing the results on the real data (Fig. 2) with the simulated data (Supplementary Fig. S2), one is able to visually assess how closely the assumptions of the PSD model resemble real datasets. When structure was simulated that differed substantially from the assumptions of the PSD model, our estimation methods were able to capture that structure just as well (Supplementary Fig. S3). This demonstrates the flexibility of the proposed approaches.

We also compared PCA and LFA to two methods of fitting the PSD model, ADMIXTURE (Alexander *etal.*, 2009) and fastSTRUCTURE (Raj *etal.*, 2014), by determining how well the methods estimated the individual-specific allele frequencies *π_ij_.* A subset of results is shown in Table 2 , and the full set of results is shown in Supplementary Table S1. For the real data scenarios, we simulated genotypes based on estimates of F from the four different methods, thus giving each method an opportunity to fit its own simulation. The methods were compared by computing three different error metrics with respect to the oracle F: Kullback–Leibler divergence, absolute error and root mean squared error (Supplementary materials, Section S4). PCA and LFA significantly outperformed ADMIXTURE and fastSTRUCTURE, which confirms the intuitive understanding of the differences between the models: the goal of Models 1 and 2 is to estimate the allele frequencies *π_ij_*, while the PSD model provides a probabilistic interpretation of the structure by modeling them as admixture proportions.

**Table 2.** A comparison of accuracy in estimating *π_ij_* parameters where data were simulated from the PSD model for varying *α*

	PCA	LFA	ADX	FS
α=0.01	$7.2\times 10^{-3}$	$7.6\times 10^{-3}$	$1.7\times 10^{-1}$	$1.7\times 10^{-1}$
α=0.1	$7.2\times 10^{-3}$	$9.3\times 10^{-3}$	$2.4\times 10^{-1}$	$2.4\times 10^{-1}$
α=0.5	$7.3\times 10^{-3}$	$9.0\times 10^{-3}$	$1.8\times 10^{-1}$	$1.8\times 10^{-1}$
α=1.0	$7.4\times 10^{-3}$	$8.4\times 10^{-3}$	$2.2\times 10^{-1}$	$2.2\times 10^{-1}$

Methods used are the proposed PCA-based method (Algorithm 1) and LFA method (Algorithms 2 and 3), and two competing methods, ADMIXTURE (ADX) and fastSTRUCTURE (FS), that directly fit the PSD model. The values reported are root mean squared error in the *π_ij_* parameter. See Supplementary Table S1 for more extensive comparisons

The computational time required to perform the proposed methods was also significantly better than ADMIXTURE and fastSTRUCTURE. Both proposed methods completed calculations on average over 10 times faster than ADMIXTURE and fastSTRUCTURE, with some scenarios as high as 150 times faster. This is notable in that both ADMIXTURE and fastSTRUCTURE are described as computationally efficient implementations of methods to estimate the PSD model (Alexander *etal.*, 2009; Raj *etal.*, 2014).

### 3.2 Analysis of the HGDP and TGP data

We analyzed the HGDP and TGP data using the proposed methods. The HGDP data consisted of *n* = 940 individuals and *m* = 431 345 SNPs, and the TGP data consisted of *n* = 1500 and *m* = 339 100 (see Supplementary materials, Section S1 for details). We first applied PCA and LFA to these datasets and made bi-plots of the top three PCs and top three LFs (Fig. 2). It can be seen that PCA and LFA provide similar visualizations of the structure present in these data. In addition, the structures estimated by these methods are related, but not identical, to the population labels provided in the original studies. We next chose a dimension *d* for the LFA model (Model 2) for each dataset. This was done by identifying the value of *d* that provides the best overall goodness of fit (Supplementary materials, Section S2). We identified *d* = 15 for HGDP and *d* = 7 for TGP based on this criterion.

One drawback of utilizing a PCA-based approach (Algorithm 1) for estimating the individual-specific allele frequencies F is that we are not guaranteed that all values of the estimates lie in [0,1], so some form of truncation is necessary. We found that 65.4% of the SNPs in the HGDP dataset and 26.5% in the TGP dataset resulted in at least one estimated individual-specific allele frequency <0 or >1 before the truncation was applied. Therefore, the truncation in forming the estimate $\tilde{F}$ is necessary when employing Algorithm 1 to estimate F from Model 1. On the other hand, due to the formulation of Model 2, all estimated allele frequencies fall in the valid range when applying LFA (Algorithms 2 and 3).

The LFA framework provides a natural computational method for ranking SNPs according to how differentiated they are with respect to structure. Accurately ranking SNPs according to this differentiation is a technique often used to identify genetic polymorphisms that are strong candidates for instances of positive selection (Coop *etal.*, 2009). Note that existing methods typically require one to first assign each individual to one of *K* discrete subpopulations (as done in Coop *etal.*, 2009) which may make unnecessary assumptions on modern datasets such as HGDP and TGP. In order to rank SNPs for differentiation, we calculate the deviance statistic when performing a logistic regression of the SNPs genotypes on the logistic factors. Specifically, we calculated the deviance by comparing the models $\text{logit}(\pi_{i})=a_{id}h_{d}$ versus $\text{logit}(\pi_{i})=\sum_{k=1}^{d}a_{ik}h_{k}$, where the former model is intercept only (i.e. *d* = 1, no structure).

Our application of LFA to identify SNPs with allele frequencies differentiated according to structure can be developed further. First, the recently proposed ‘jackstraw’ approach (Chung and Storey, 2015) provides a manner in which statistical significance can be assigned to these SNPs. Assigning statistical significance to the population differentiation of SNPs has traditionally been a difficult problem (Akey *etal.*, 2002). Second, we found the deviance measure tends to have more extreme values for SNPs with larger minor allele frequencies (MAFs). Therefore, the ranking of SNPs may be made more informative if MAF is taken into account. Third, although this ranking is identifying differentiation and not specifically selection, it may provide a useful starting point in understanding methods that attempt to detect selection.

**Fig. 3.**
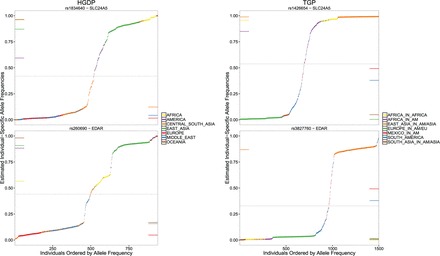
SNPs with highly differentiated allele frequencies with respect to structure. Two of the most highly different SNPs according to LFA are shown for the HGDP and TGP datasets. For each SNP, the ${\hat{\pi }}_{ij}$ values are ordered and they are colored according reported ancestry. The horizontal bars on the sides of the plots denote the usual allele frequency estimates formed within each ancestral group

The most differentiated SNPs (Supplementary Tables S2 and Supplementary Data) reveal some noteworthy results, especially considering the flexible approach to forming the ranking. SNPs located within or very close to *SLC24A5* were the top ranked in both HGDP and TGP. This gene is well known to be involved in determining skin pigmentation in humans (Lamason *etal.*, 2005) and is hypothesized to have been subject to positive selection (Sabeti *etal.*, 2007). The next most highly ranked SNPs in both studies are located in *EDAR*, which plays a major role in distinguishing phenotypes (e.g. hair follicles) among Asians. SNP rs3827760 is the second most differentiated SNP in the TGP data, which has also been hypothesized to be under positive selection in humans and whose causal role in the hair follicle phenotype has been verified in a mouse model (Kamberov *etal.*, 2013). SNPs corresponding to these two genes for both studies are plotted in increasing order of ${\hat{\pi }}_{ij}$ values, revealing subtle variation within each major ancestral group in addition to coarser differences in allele frequency (Fig. 3). Other noteworthy genes with highly differentiated proximal SNPs include: We have provided information on the 5000 most differentiated SNPs for both TGP and HGDP as Supplementary material files.

- *FOXP1*, which is a candidate gene for involvement in tumor progression and plays an important regulatory role with *FOXP2* (Banham *etal.*, 2001; Shigekawa *etal.*, 2011);
- *TBC1D1* in which genetic variation has been shown to confer risk for severe obesity in females (Stone *etal.*, 2006);
- *KIF3C*, a novel kinesin-like protein, which has been hypothesized to be involved in microtubule-based transport in neuronal cells (Sardella *etal.*, 1998);
- *KCNMA1*, a recently identified susceptibility locus for obesity (Jiao *etal.*, 2011);
- *CTNNA3* in which genetic variation has been shown to be associated with diisocyanate-induced occupational asthma (Bernstein *etal.*, 2013);
- *PTK6*, breast tumor kinase (Brk), which is known to function in cell-type and context-dependent processes governing normal differentiation (Ostrander *etal.*, 2010).

## 4 Discussion

We have investigated two latent variable models of population structure to simultaneously estimate all individual-specific allele frequencies from genome-wide genotyping data. Model 1, a direct model of allele frequencies, can be estimated by using a modified PCA and Model 2, a model of the logit transformation of allele frequencies, is estimated through a new approach we called LFA. For both models, the latent variables are estimated in a non-parametric fashion, meaning we do not make any assumptions about the underlying structure captured by the latent variables. These models are general in that they allow for each individual’s genotype to be generated from an allele frequency specific to that individual, which includes discretely structured populations, admixed populations and spatially structured populations. In LFA, we construct a model of the logit of these allele frequencies in terms of underlying factors that capture the population structure. We have proposed a computationally efficient method to estimate this model that requires only two applications of SVD. This approach builds on the success of PCA in that we are able to capture population structure in terms of a low-dimensional basis. It improves on PCA in that the latent variables we estimate can be straightforwardly incorporated into downstream statistical inference procedures that require well-behaved estimates of allele frequencies. In particular, statistical inferences of Hardy–Weinberg equilibrium, $F_{\text{ST}}$, and marker-trait associations are amenable to complex population structures within our framework.

We demonstrated our proposed approach on the HGDP and TGP datasets and several simulated datasets motivated by the HapMap, HGDP and TGP datasets as well as the PSD model and spatially distributed structures. It was shown that our method estimates the underlying logistic factors with a high degree of accuracy. We also showed that applying PCA to genotype data estimates a row basis of population structure on the original allele frequency scale to a high degree of accuracy. However, problems occur when trying to recover estimates of individual-specific allele frequencies because PCA is a real-valued model that does not always result in allele frequency estimates lying between 0 and 1.

Although PCA has become very popular for genome-wide genotype data, it should be stressed that PCA is fundamentally a method for characterizing variance and special care should be taken when applying it to estimate latent variables. The authoritative treatment of PCA (Jolliffe, 2010) eloquently makes this point throughout the text and considers cases where factor analysis is more appropriate than PCA through examples reminiscent of the population structure problem. Here, we have shown that modeling and estimating population structure can be understood from the factor analysis perspective, leading to estimates of individual-specific allele frequencies through their natural parameter on the logit scale. At the same time, we have avoided some of the difficulties of traditional parametric factor analysis by maintaining the relevant non-parametric properties of PCA, specifically in making no assumptions about the underlying probability distributions of the logistic factors that capture population structure.

## Supplementary Material

Supplementary Data

## References

[btv641-B1] 1000 Genomes Project Consortium (2010) A map of human genome variation from population-scale sequencing. Nature, 467, 1061–1073. 20981092 10.1038/nature09534 PMC3042601

[btv641-B2] Akey J.M. (2002) Interrogating a high-density SNP map for signatures of natural selection. Genome Res., 12, 1805–1814. 12466284 10.1101/gr.631202 PMC187574

[btv641-B3] Alexander D.H. (2009) Fast model-based estimation of ancestry in unrelated individuals. Genome Res., 19, 1655–1664. 19648217 10.1101/gr.094052.109 PMC2752134

[btv641-B4] Astle W. Balding D.J. (2009) Population structure and cryptic relatedness in genetic association studies. Stat. Sci., 24, 451–471.

[btv641-B5] Baglama J. Reichel L. (2006) Restarted block Lanczos Bidiagonalization methods. Numer. Algorithms, 43, 251–272.

[btv641-B6] Balding D.J. Nichols R.A. (1995) A method for quantifying differentiation between populations at multi-allelic loci and its implications for investigating identity and paternity. Genetica, 96, 3–12. 7607457 10.1007/BF01441146

[btv641-B7] Banham A.H. (2001) The foxp1 winged helix transcription factor is a novel candidate tumor suppressor gene on chromosome 3p. Cancer Res., 61, 8820–8829. 11751404

[btv641-B8] Bartholomew D.J. (2011) Latent Variable Models and Factor Analysis: A Unified Approach. Wiley Series in Probability and Statistics.

[btv641-B9] Bernstein D.I. (2013) Ctnna3 (*α*-catenin) gene variants are associated with diisocyanate asthma: a replication study in a Caucasian worker population. Toxicol. Sci.*, *131, 242–246. 22977168 10.1093/toxsci/kfs272 PMC3537126

[btv641-B10] Cann H.M. (2002) A human genome diversity cell line panel. Science, 296, 261–262. 11954565 10.1126/science.296.5566.261b

[btv641-B11] Chung N.C. Storey J.D. (2015) Statistical significance of variables driving systematic variation. Bioinformatics, 31, 545–554. 25336500 10.1093/bioinformatics/btu674 PMC4325543

[btv641-B12] Collins M. (2002) A generalization of principal component analysis to the exponential family. In: Dietterich,T.G. *et al*. (eds), Proceedings of Advances in Neural Information Processing Systems (NIPS), MIT Press, vol. 14, pp. 617–624.

[btv641-B13] Coop G. (2009) The role of geography in human adaptation. PLoS Genet., 5, e1000500. 19503611 10.1371/journal.pgen.1000500 PMC2685456

[btv641-B14] Corona E. (2013) Analysis of the genetic basis of disease in the context of worldwide human relationships and migration. PLoS Genet., 9, e1003447. 23717210 10.1371/journal.pgen.1003447 PMC3662561

[btv641-B15] Engelhardt B.E. Stephens M. (2010) Analysis of population structure: a unifying framework and novel methods based on sparse factor analysis. PLoS Genet., 6, e1001117. 20862358 10.1371/journal.pgen.1001117 PMC2940725

[btv641-B16] Jiao H. (2011) Genome wide association study identifies kcnma1 contributing to human obesity. BMC Med. Genom., 4, 51. 10.1186/1755-8794-4-51 PMC3148553 21708048

[btv641-B17] Jolliffe I.T. (2010) Principal Component Analysis, 2nd edn Springer, New York.

[btv641-B18] Kamberov Y. (2013) Modeling recent human evolution in mice by expression of a selected EDAR variant. Cell, 152, 691–702. 23415220 10.1016/j.cell.2013.01.016 PMC3575602

[btv641-B19] Lamason R.L. (2005) Slc24a5, a putative cation exchanger, affects pigmentation in zebrafish and humans. Science, 310, 1782–1786. 16357253 10.1126/science.1116238

[btv641-B20] Lehmann E.L. Casella G. (1998) Theory of Point Estimation, 2nd edn Springer, New York.,

[btv641-B21] Manni F. (2010). Interview with Luigi Luca Cavalli-Sforza: past research and directions for future investigations in human population genetics. Hum. Biol., 82, 245–266. 20649383 10.3378/027.082.0301

[btv641-B22] McCullagh P. Nelder J.A. (1989) Generalized Linear Models. Chapman and Hall, London.

[btv641-B23] Menozzi P. (1978) Synthetic maps of human gene frequencies in Europeans. Science, 201, 786–792. 356262 10.1126/science.356262

[btv641-B24] Novembre J. Stephens M. (2008) Interpreting principal component analyses of spatial population genetic variation. Nat. Genet., 40, 646–649. 18425127 10.1038/ng.139 PMC3989108

[btv641-B25] Ostrander J.H. (2010) Brk/ptk6 signaling in normal and cancer cell models. Curr. Opin. Pharmacol., 10, 662–669. 20832360 10.1016/j.coph.2010.08.007 PMC2981671

[btv641-B26] Pritchard J.K. (2000) Inference of population structure using multilocus genotype data. Genetics, 155, 945–959. 10835412 10.1093/genetics/155.2.945 PMC1461096

[btv641-B27] Raj A. (2014) fastSTRUCTURE: Variational inference of population structure in large SNP datasets. Genetics, 197, 573–589. 24700103 10.1534/genetics.114.164350 PMC4063916

[btv641-B28] Rendine S. (1999) A problem with synthetic maps: Reply to Sokal et al. Hum. Biol., 71, 15–25. 9972095

[btv641-B29] Rosenberg N.A. (2002) Genetic structure of human populations. Science, 298, 2381–2385. 12493913 10.1126/science.1078311

[btv641-B30] Rosenberg N.A. (2005) Clines, clusters, and the effect of study design on the inference of human population structure. PLoS Genet., 1, e70. 16355252 10.1371/journal.pgen.0010070 PMC1310579

[btv641-B31] Sabeti P.C. (2007) Genome-wide detection and characterization of positive selection in human populations. Nature, 449, 913–918. 17943131 10.1038/nature06250 PMC2687721

[btv641-B32] Sardella M. (1998) Kif3c, a novel member of the kinesin superfamily: sequence, expression, and mapping to human chromosome 2 at 2p23. Genomics, 47, 405–408. 9480755 10.1006/geno.1997.5123

[btv641-B33] Shigekawa T. (2011) Foxp1, an estrogen-inducible transcription factor, modulates cell proliferation in breast cancer cells and 5-year recurrence-free survival of patients with tamoxifen-treated breast cancer. Hormon. Cancer, 2, 286–297. 10.1007/s12672-011-0082-6 PMC10358050 21901488

[btv641-B34] Sokal R.R. (1999). A problem with synthetic maps. Hum. Biol., 71, 1–13. 9972095

[btv641-B35] Song M. (2015) Testing for genetic associations in arbitrarily structured populations. Nat. Genet., 47, 550–554. 25822090 10.1038/ng.3244 PMC4464830

[btv641-B36] Stone S. (2006) Tbc1d1 is a candidate for a severe obesity gene and evidence for a gene/gene interaction in obesity predisposition. Hum. Mol. Genet., 15, 2709–2720. 16893906 10.1093/hmg/ddl204

[btv641-B37] Tang H. (2005) Estimation of individual admixture: analytical and study design considerations. Genet. Epidemiol., 28, 289–301. 15712363 10.1002/gepi.20064

[btv641-B38] Thornton T. (2012) Estimating kinship in admixed populations. Am. J. Hum. Genet., 91, 122–138. 22748210 10.1016/j.ajhg.2012.05.024 PMC3397261

