# Supplementary material for: Probabilistic models of genetic variation in structured populations applied to global human studies
Source: Bioinformatics. 2015 Nov 6;32(5):713–21. doi: 10.1093/bioinformatics/btv641 (PMC4795615; doi:10.1093/bioinformatics/btv641)
Supplement: Supplementary Data [file btv641_supplementary_data.zip › btv641_supplementary.pdf]

Supplementary Materials:
Probabilistic models of genetic variation in structured populations applied to global human studies

Wei Hao[1*], Minsun Song[1*+], and John D. Storey[1,2 †]

1. Lewis-Sigler Institute for Integrative Genomics, Princeton University, Princeton, NJ 08544.

2. Center for Statistics and Machine Learning, Princeton University, Princeton, NJ 08544.

[*] These authors contributed equally to this work.

[+] Present address: Department of Mathematics and Statistics, University of Nevada Reno, Reno, NV, 89557.

[†] To whom correspondence should be addressed: jstorey@princeton.edu

SUPPLEMENTARY TEXT

S1 Data sets

The HGDP data set was constructed by intersecting the data available from the HGDP web site, http://www.hagsc.org/hgdp/files.html, with the set of individuals "H952" identified by Rosenberg (2006) [1] with a high confidence as containing no first and second-degree relative pairs. This yielded complete SNP genotype data on 431,345 SNPs for 940 individuals.

In order to obtain data from the TGP we first obtained the genotype data that had been measured through the Omni Platform, 2011-11-17, ftp://ftp.1000genomes.ebi.ac.uk/vol1/ftp/technical/working. We removed related individuals based on the TGP sample information. We then sorted individuals according to least percentage of SNPs with missing data, and we selected the top 1500 individuals. This yielded complete SNP genotype data on 339,100 SNPs for 1500 individuals.

We utilized the HapMap data set in the simulated data described below. We obtained the HapMap data release 23a, NCBI build 36 from www.hapmap.org consisting of unrelated individuals: 60 from European ancestry group (CEU), 60 from Yoruba, Africa (YRI) , and 90 from Japan and China (JPT+CHB). We identified all SNPs with observed minor allele frequency $\geq 5\%$ and with no missing data. The total number of SNPs used after filtering in each population were CEU: 1,416,940, YRI: 1,539,314, JPT+CHB: 759,452. We then identified all SNPs common to all three populations resulting in a total of 363,955.

S2 Choosing the model dimension

The model dimension d was determined for the HGDP and TGP data sets under the rationale that when d is large enough, then the model should fit a great majority of the SNPs. When d is too small, then the structure which has not been accounted for will lead to spurious deviations. Values $d = 1, 2, \ldots, 20$ were considered for each data set, and we ended up identifying $d = 15$ for HGDP and $d = 7$ for TGP. We note that these choices could also be interpreted as reasonable according to a scree plot when PCA was applied to the genotype data.

For a given d value, we formed $\widehat{\mathbf{F}}$ using the LFA method. We calculated a goodness-of-fit statistic for each SNP i as follows:

$$\sum_{k=0}^{2} \frac{\left[\sum_{j=1}^{n} 1(x_{ij} = k) - \sum_{j=1}^{n} \binom{2}{k} \widehat{\pi}_{ij}^{k} (1 - \widehat{\pi}_{ij})^{2-k}\right]^2}{\sum_{j=1}^{n} \binom{2}{k} \widehat{\pi}_{ij}^{k} (1 - \widehat{\pi}_{ij})^{2-k}},$$

where $\sum_{j=1}^{n} 1(x_{ij} = k)$ is the observed number of genotypes equal to k and $\sum_{j=1}^{n} \binom{2}{k} \widehat{\pi}_{ij}^{k} (1 - \widehat{\pi}_{ij})^{2-k}$ is the expected number of genotypes equal to k. We then utilized $\widehat{\mathbf{F}}$ to simulate five instances of a genotype matrix $\mathbf{X}^0$, assuming the LFA model, where we simulated $x_{ij}^0 \sim$ Binomial$(2, \widehat{\pi}_{ij})$. On each simulated genotype matrix $\mathbf{X}^0$, we again applied LFA to obtain $\widehat{\mathbf{F}}^0$ and calculate goodness-of-fit statistics. These goodness-of-fit statistics were then pooled across all five simulated data sets and across all SNPs to form the null distribution, which then allowed us to calculate a goodness-of-fit p-value for each observed SNP. (It should be noted that we also formed a separate null distribution according to minor allele frequency bins of length 0.05, and we arrived at the same conclusion.) We then compared these p-values to the Uniform(0,1) distribution and also against the p-values from the $d + 1$ case. This allowed us to identify a value of d where the goodness-of-fit p-values were both close to the Uniform(0,1) distribution and to the goodness-of-fit p-values from the $d + 1$ case.

S3 Simulated data

For each simulation scenario, genotypes $\mathbf{X}$ were simulated such that $x_{ij} \sim$ Binomial$(2, \pi_{ij})$, where π_{ij} were elements of the allele frequency matrix $\mathbf{F}$. The results from the simulated data are summarized in Tables 1 and 2.

Balding-Nichols (BN). For each SNP in the HapMap data set, we estimated its marginal allele frequency according to the observed frequency and estimated its $\mathrm{F_{ST}}$ value using the Weir & Cockerham estimate [2]. We set the simulated data to have $m = 100,000$ SNPs and $n = 5000$ individuals with $d = 3$. Using Model 1, the $\mathbf{S}$ matrix was generated by sampling its columns $\mathbf{s}^j$ i.i.d. from $(1, 0, 0)^T$, $(0, 1, 0)^T$, and $(0, 0, 1)^T$ with respective probabilities $60/210$, $60/210$, and $90/210$ to reflect the original data's

subpopulation proportions. For each row i of $\mathbf{\Gamma}$, we simulated i.i.d. draws from the Balding-Nichols model: $\gamma_{i1}, \gamma_{i2}, \gamma_{i3} \stackrel{i.i.d.}{\sim}$ BN(p_i, F_i), where the pair (p_i, F_i) was randomly selected from among the marginal allele frequency and $\mathrm{F_{ST}}$ pairs calculated on the HapMap data set.

PSD. We analyzed each SNP in the HGDP data set to estimate its marginal allele frequency according to the observed marginal frequency and $\mathrm{F_{ST}}$ using the Weir & Cockerham estimate [2]. To estimate $\mathrm{F_{ST}}$, each individual in the HGDP data set was assigned to one of $K = 5$ subpopulations according to the analysis in Rosenberg et al. (2002) [3]. We set $m = 100,000$ SNPs and $n = 5000$ individuals with $d = 3$. Again utilizing Model 1, each row i of $\mathbf{\Gamma}$ was simulated according to $\gamma_{i1}, \gamma_{i2}, \gamma_{i3} \stackrel{i.i.d.}{\sim}$ BN(p_i, F_i), where the pair (p_i, F_i) was randomly selected from among the marginal allele frequency and $\mathrm{F_{ST}}$ pairs calculated on the HGDP data set. To generate $\mathbf{S}$, we simulated $(s_{1j}, s_{2j}, s_{3j}) \stackrel{i.i.d.}{\sim}$ Dirichlet$(\boldsymbol{\alpha})$ for $j = 1, \ldots, 5000$. We considered $\boldsymbol{\alpha} = (0.01, 0.01, 0.01)$, $\boldsymbol{\alpha} = (0.1, 0.1, 0.1)$, $\boldsymbol{\alpha} = (0.5, 0.5, 0.5)$, and $\boldsymbol{\alpha} = (1, 1, 1)$. It should be noted that as $\boldsymbol{\alpha} \to \mathbf{0}$, the draws from the Dirichlet distribution become increasingly closer to assigning each individual to one of three discrete subpopulations with equal probability. When $\boldsymbol{\alpha} = (1, 1, 1)$, the admixture proportions are distributed uniformly over the simplex.

Spatial. This scenario is meant to create population structure that is driven by spatial position of the individual. We set the simulated data to have $m = 100,000$ SNPs and $n = 5000$ individuals with $d = 3$. Rows $i = 1, 2$ of $\mathbf{S}$ were simulated as $s_{ij} \stackrel{i.i.d.}{\sim}$ Beta(a, a) for $j = 1, \ldots, 5000$, and row 3 of $\mathbf{S}$ contained the intercept term, $s_{3j} = 1$. We considered four values of a: 0.1, 0.25, 0.5, and 1. The first two rows of $\mathbf{S}$ place each individual in a two-dimensional space (Figure S3), where the ancestry of individual j is located at (s_{1j}, s_{2j}) in the unit square. When $a = 1$, the Beta(a, a) distribution is Uniform$(0, 1)$, so this scenario represents a uniform distribution of individuals in unit square. As $a \to 0$, the Beta(a, a) places each individual with equal probabilities in one of the four corners of the unit square. The matrix $\mathbf{\Gamma}$ was created by sampling $\gamma_{ij} \stackrel{i.i.d.}{\sim} 0.9 \times$ Uniform$(0, 1/2)$ for $j = 1, 2$ and $\gamma_{i3} = 0.05$. It should be noted that all $\pi_{ij} \in [0.05, 0.95]$ by construction.

Real Data. For the HGDP and TGP scenarios, we estimated an allele frequency matrix $\mathbf{F}$ from the real data via four different methods. For HGDP we had $m = 431,345$ SNPs by $n = 940$ individuals with $d = 15$, and for TGP we had $m = 339,100$ and $n = 1,500$ with $d = 7$. The four methods are:

- *PCA*: $\mathbf{F}$ was taken to be the matrix $\widetilde{\mathbf{F}}$ estimated via Algorithm 1.
- *LFA*: $\mathbf{F} = \mathrm{logit}^{-1}(\widehat{\mathbf{L}})$, where $\widehat{\mathbf{L}}$ was estimated via Algorithm 3.
- *ADX*: $\mathbf{F}$ was taken to be the matrix formed by computing the marginal allele frequencies in the Pritchard-Stephens-Donnelly model, i.e. $\mathbf{F} = \mathbf{PQ}$, and $\mathbf{P}$ and $\mathbf{Q}$ were estimated via the software ADMIXTURE [4].
- *FS*: Same as above except $\mathbf{P}$ and $\mathbf{Q}$ are estimated via the software fastSTRUCTURE [5].

S4 Error Measures Used to Evaluate Estimates of $\mathbf{F}$ and $\mathbf{L}$

Estimates of π_{ij} were evaluated with three different metrics. Let $\widehat{\pi}_{ij}$ be the estimate for any given method.

The *Kullback-Leibler divergence* for the binomial distribution allows us to measure the difference between the distribution from the estimated allele frequencies to the distribution from the oracle allele frequencies:

$$\text{KL} = \pi_{ij} \ln\left(\frac{\pi_{ij}}{\widehat{\pi}_{ij}}\right) + (1-\pi_{ij}) \ln\left(\frac{1-\pi_{ij}}{1-\widehat{\pi}_{ij}}\right).$$

Mean absolute error compares the allele frequencies directly:

$$\text{MAE} = \frac{1}{m \times n} \sum_{i=1}^{m} \sum_{j=1}^{n} \left| \pi_{ij} - \widehat{\pi}_{ij} \right|.$$

Root mean squared error:

$$\text{RMSE} = \sqrt{\frac{1}{m \times n} \sum_{i=1}^{m} \sum_{j=1}^{n} (\text{logit}(\pi_{ij}) - \text{logit}(\widehat{\pi}_{ij}))^2}.$$

S5 $\mathrm{F_{ST}}$ for individual-specific allele frequencies

By considering the derivation of $\mathrm{F_{ST}}$ for K discrete populations as described in Weir (1984, 1996) [2,6], it can be seen that a potential generalization of $\mathrm{F_{ST}}$ to arbitrary population structure is

$$\mathrm{F_{ST}} = 1 - \frac{\mathrm{E}_{\boldsymbol{Z}}[\mathrm{Var}(x|\boldsymbol{Z})]}{\mathrm{Var}(x)},$$

where, as described in Section 2.1, $\boldsymbol{Z}$ is a latent variable capturing an individual's population structure position or membership. The allele frequency of a SNP conditional on $\boldsymbol{Z}$ can be viewed as being a function of $\boldsymbol{Z}$, which we have denoted by $\pi(\boldsymbol{Z})$. If n individuals are sampled independently and homogeneously from the population[1] such that $\boldsymbol{z}_1, \ldots, \boldsymbol{z}_n$ are i.i.d. from the distribution on $\boldsymbol{Z}$, then for SNP i that satisfies the model assumptions, it follows that $\mathrm{Var}(x_{ij}|z_j) = 2\pi_{ij}(1-\pi_{ij})$ and

$$\mathrm{F_{ST}} \overset{a.s.}{=} \lim_{n \to \infty} 1 - \frac{\frac{1}{n}\sum_{j=1}^{n} \pi_{ij}(1-\pi_{ij})}{\overline{\pi}_i(1-\overline{\pi}_i)},$$

[1]When the individuals are not sampled homogeneously throughout the population (e.g., in the HapMap data with 60, 60, and 90 observations from three discretely defined subpopulations), then it may be the case that the above quantity should be modified to reflect the stratified or non-homogeneous sampling.

where $\overline{\pi}_i = \sum_{j=1}^{n} \pi_{ij}/n$ is the marginal allele frequency among the n individuals. Thus, good estimates of the π_{ij} values may be useful for estimating $\mathrm{F_{ST}}$ in this general setting. One example would be to form a plug-in estimate of $\mathrm{F_{ST}}$ by replacing π_{ij} with $\widehat{\pi}_{ij}$ from the proposed LFA method.

S6 Relationship of LFA to existing models and methods

The problem of modeling a genotype matrix $\mathbf{X}$ in order to uncover latent variables that explain cryptic structure is a special case of a much more general problem that has been considered for several years in the statistics literature [7, 8]. Under a latent variable model, it is assumed that the "manifest" (observed) variables are the result of the "latent" (unobserved) variables. Different types of the latent variable models can be grouped according to whether the manifest and latent variables are categorical or continuous. For example, factor analysis is a latent variable method for the case where both manifest variable and latent variable are continuous. A proposed naming convention [9] is summarized as follows:

	Manifest variables	
Latent variables	Continuous	Categorical
Continuous	Factor analysis	Latent trait analysis
Categorical	Latent profile analysis	Latent class analysis

The problem we consider is that the manifest variables (observed gentoypes) are categorical, and they are driven by latent variables (population structure) that may either be categorical (discrete population structure) or continuous (complex population structure). Therefore, the LFA method may be described as a nonparametric latent variable estimation method that jointly captures latent trait analysis and latent class analysis. Another naming convention that we could apply to LFA would be to call it a nonparametric latent variable model for Binomial data. The naming conventions of latent variable models are inconsistent and often confusing [9].

Bartholomew (1980) [10] proposed a model related to equation (2) to identify latent variables that influence the probabilities of a collection of Binomial random variables. See also Bartholomew et al. 2011 for a comprehensive treatment of this area, which they call "general linear latent variable models" (GLLVM). In particular, when the manifest variables $x_{ij} \sim$ Bernoulli(π_{ij}) and the latent variables h_{kj} are continuous variables, the GLLVM in this case is Model 2 , $\mathrm{logit}(\pi_{ij}) = \sum_{k=1}^{d} a_{ik} h_{kj}$. While we begin with this model, there are some key differences. The number of manifest variables in the data considered in Bartholomew (1980) and related work is notably smaller than genome-wide genotype data, so the assumptions and estimation approach differ substantially. Model assumptions are typically made about the probability distributions of the latent variables; we consider these model assumptions

too strong and also unnecessary for the genome-wide genotype data considered here, although they may be quite reasonable for the problems considered in other contexts. Existing methods typically estimate Model 2 by calculating the joint posterior distribution of the h_{kj} based on an assumed prior distribution of the latent variables.

Our LFA approach for estimating the row basis of $\mathbf{L}$ is nonparametric since it does not require a prior assumption on the distribution of latent variables, $\mathbf{H}$. The model fitting methods of ref. [9] are too computationally intensive for high-dimensional data, requiring many iterations and potential convergence issues. Our proposed algorithm requires performing SVD twice, which leads to a dramatic reduction in computational burden and difficulties. Engelhardt and Stephens (2010) [11] make an interesting connection between classical factor analysis models of $\mathbf{F}$ and other models of population structure, but the factor analysis model runs into the difficulty that the latent factors are assumed to be Normal distributed, and the constraint that alleles frequencies are in $[0, 1]$ is not easily accommodated by this continuous, real-valued model.

Several extensions of PCA to categorical data have been proposed [12–14]. We found that the algorithms perform very slowly on genome-wide genotyping data, and the estimation can be quite poor when $d > 1$. Also, PCA is essentially a method for characterizing variance in data [15], and the latent variable approach is more directly aimed at uncovering latent population structure. Non-negative matrix factorization (NMF) [16] is another matrix factorization for count data (e.g., Poisson random variables). This identifies two non-negative matrices whose product approximates the original matrix. However, similarly to PCA, we do not find that this approach easily translates into interpretable models of population and it is computationally intensive. NMF has proven to be quite useful as a numerical tool for decomposing images into parts humans recognize as distinct [17].

References

[1] Rosenberg, N. A. Standardized subsets of the hgdp-ceph human genome diversity cell line panel, accounting for atypical and duplicated samples and pairs of close relatives. *Annals of Human Genetics* **70**, 841–847 (2006).

[2] Weir, B. and Cockerham, C. Estimating F-statistics for the analysis of population structure. *Evolution* **38**, 1358–1370 (1984).

[3] Rosenberg, N. A., Pritchard, J. K., Weber, J. L., Cann, H. M., Kidd, K. K., Zhivotovsky, L. A., and Feldman, M. W. Genetic structure of human populations. *Science* **298**, 2381–2385 (2002).

[4] Alexander, D. H., Novembre, J., and Lange, K. Fast model-based estimation of ancestry in unrelated individuals. *Genome Research* **19**(9), 1655–1664 (2009).

[5] Raj, A., Stephens, M., and Pritchard, J. K. fastSTRUCTURE: Variational inference of population structure in large snp datasets. *Genetics* **197**, 573–589 (2014).

[6] Weir, B. S. *Genetic Data Analysis II: Methods for Discrete Population Genetic Data*. Sunderland, MA: Sinauer Associates, (1996).

[7] Bartholomew, D. J. The foundations of factor analysis. *Biometrika* **71**, 221–232 (1984).

[8] Moustaki and Knott. Generalized latent trait models. *Psychometrika* **65**, 391–411 (2000).

[9] Bartholomew, D. J., Knott, M., and Moustaki, I. *Latent Variable Models and Factor Analysis: A Unified Approach*. Wiley Series in Probability and Statistics, (2011).

[10] Bartholomew, D. J. Factor analysis for categorical data. *J Roy Stat Soc B* **42**, 293–321 (1980).

[11] Engelhardt, B. E. and Stephens, M. Analysis of population structure: a unifying framework and novel methods based on sparse factor analysis. *PLoS Genet* **6**(9) (2010).

[12] Collins, M., Dasgupta, S., and Schapire, R. A generalization of principle component analysis to the exponential family. In *Proceedings of Advances in Neural Information Processiong Systems*, (2002).

[13] Schein, A. I., Saul, L. K., and Ungar, L. H. A generalized linear model for principal component analysis of binary data. In *Proceedings of the 9 th International Workshop on Artificial Intelligence and Statistics*, (2003).

[14] Guo, Y. and Schuurmans, D. Efficient global optimization for exponential family pca and low-rank matrix factorization. In *In Allerton Conf. on Commun., Control, and Computing*, (2008).

[15] Jolliffe, I. T. *Principal component analysis*. New York: Springer, 2nd edition, (2010).

[16] Paatero, P. and Tapper, U. Positive matrix factorization: A non-negative factor model with optimal utilization of error estimates of data values. *Environmetrics* **5**, 111–126 (1994).

[17] Lee, D. D. and Seung, S. Learning the parts of objects by non-negative matrix factorization. *Nature* **401**, 788–791 (1999).

SUPPLEMENTARY FIGURES AND TABLES

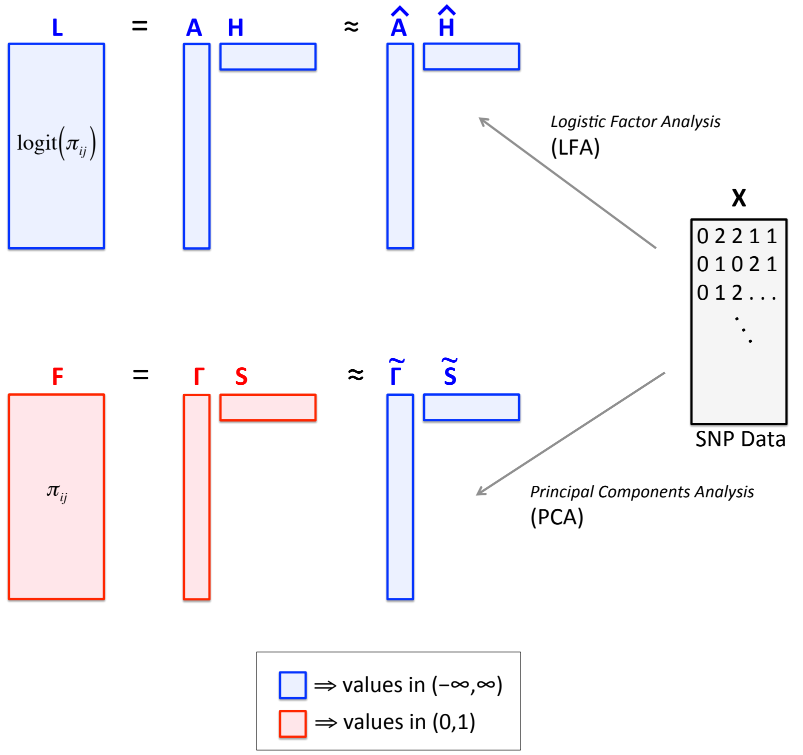

Figure S1: A comparison of LFA model (2) and its estimate to model (1) and its PCA estimate. The proposed LFA approach first models the logit of the individual-specific allele frequencies in terms of the product of two matrices, the left matrix establishing how population structure is present in allele frequencies, and the right matrix giving the structure. Whereas the LFA approach preserves the scale of the model through the estimate (all real-valued numbers), the same is not true to PCA. This leads to issues in the estimation of individual-specific allele frequencies when utilizing PCA. We have shown, however, that PCA estimates very well a row basis for $\mathbf{S}$ from Model 1. This connects PCA to an explicit model of population structure.

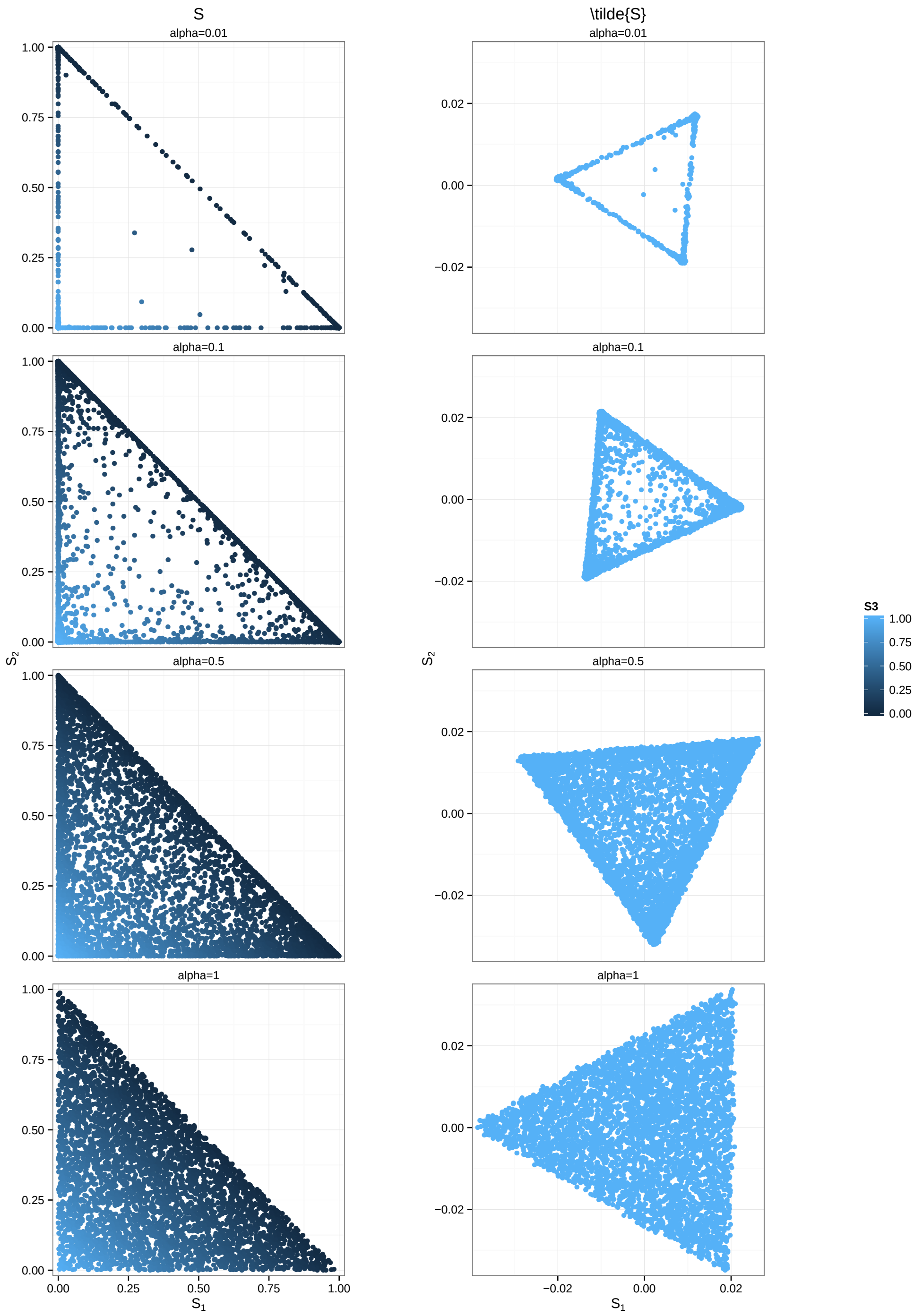

Figure S2: A mapping from $\mathbf{S}$ to $\widetilde{\mathbf{S}}$ for four simulated $\mathbf{S}$ matrices under the PSD model. The left column shows the simulated structure $\mathbf{S}$ for each of four scenarios (a–d) and the right column shows the resulting estimated row basis of $\mathbf{S}$ produced from PCA. It can be seen that the scale on which $\mathbf{S}$ was generated, all values in (0,1), is lost in the principal components, values in $\mathbb{R}$.

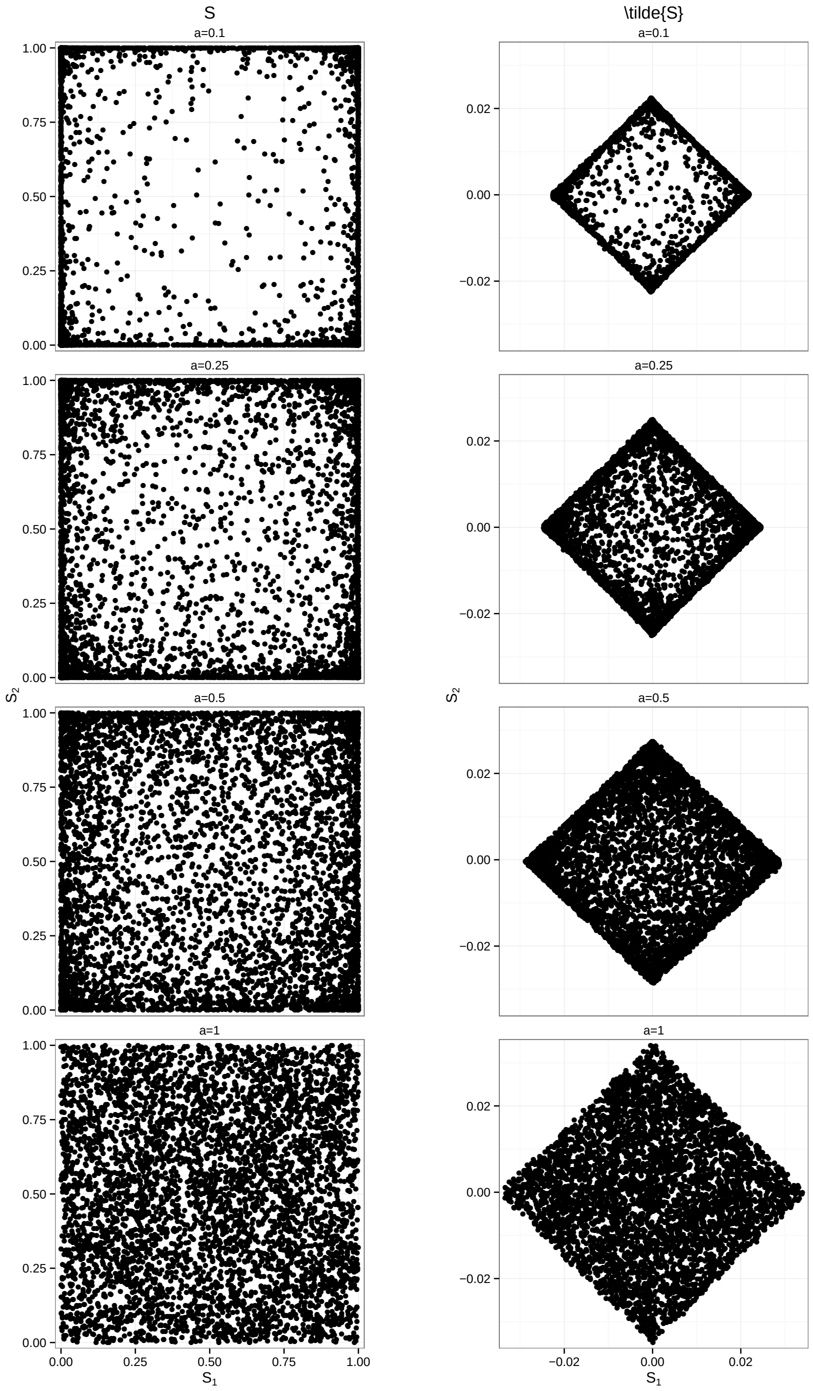

Figure S3: A mapping from $\mathbf{S}$ to $\widetilde{\mathbf{S}}$ for four simulated $\mathbf{S}$ matrices under the Spatial model. The left column shows the simulated structure $\mathbf{S}$ for each of four scenarios (a–d) and the right column shows the resulting estimated row basis of $\mathbf{S}$ produced from PCA. It can be seen that the scale on which $\mathbf{S}$ was generated, all values in (0,1), is lost in the principal components, values in $\mathbb{R}$.

Table S1: Accuracy in estimating π_{ij} parameters by the PCA based method and LFA. Each row is a different simulation scenario. Each column is the accuracy of a method's fits with the given metric.

Scenario		Median KL				Mean Abs. Err.				RMSE			
		PCA	LFA	ADX	FS	PCA	LFA	ADX	FS	PCA	LFA	ADX	FS
BN		6.9E-5	6.8E-5	2.6E-3	2.6E-3	5.8E-3	5.8E-3	3.7E-2	3.7E-2	7.5E-3	7.5E-3	5.8E-2	5.8E-2
PSD	$\alpha = 0.01$	7.0E-5	7.3E-5	1.6E-2	1.6E-2	5.6E-3	5.8E-3	9.7E-2	9.7E-2	7.2E-3	7.6E-3	1.7E-1	1.7E-1
	$\alpha = 0.1$	6.7E-5	9.2E-5	3.6E-2	3.6E-2	5.6E-3	6.9E-3	1.6E-1	1.6E-1	7.2E-3	9.3E-3	2.4E-1	2.4E-1
	$\alpha = 0.5$	6.3E-5	8.5E-5	5.4E-2	5.4E-2	5.6E-3	6.8E-3	1.4E-1	1.4E-1	7.3E-3	9.0E-3	1.8E-1	1.8E-1
	$\alpha = 1.0$	6.1E-5	7.4E-5	3.3E-2	3.3E-2	5.6E-3	6.3E-3	1.4E-1	1.4E-1	7.4E-3	8.4E-3	2.2E-1	2.2E-1
Spatial	$a = 0.1$	7.3E-5	1.2E-4	8.2E-3	8.1E-3	5.5E-3	7.6E-3	7.4E-2	7.4E-2	7.0E-3	1.0E-2	1.2E-1	1.2E-1
	$a = 0.25$	6.9E-5	1.1E-4	8.6E-3	8.6E-3	5.6E-3	7.4E-3	9.3E-2	9.3E-2	7.2E-3	9.8E-3	1.6E-1	1.6E-1
	$a = 0.5$	6.6E-5	9.5E-5	1.0E-2	1.0E-2	5.6E-3	6.9E-3	6.7E-2	6.7E-2	7.2E-3	9.2E-3	1.0E-1	1.0E-1
	$a = 1.0$	6.3E-5	7.8E-5	1.2E-2	1.2E-2	5.7E-3	6.4E-3	1.1E-1	1.1E-1	7.4E-3	8.5E-3	1.7E-1	1.7E-1
TGP fit	PCA	4.1E-4	5.2E-4	2.8E-3	3.4E-3	1.3E-2	1.5E-2	8.1E-2	8.3E-2	1.8E-2	2.1E-2	1.5E-1	1.5E-1
	LFA	4.3E-4	4.8E-4	2.4E-3	2.7E-3	1.3E-2	1.4E-2	7.9E-2	8.1E-2	1.8E-2	2.0E-2	1.4E-1	1.5E-1
	ADX	5.4E-4	4.4E-4	5.0E-3	5.5E-3	1.5E-2	1.3E-2	1.1E-1	1.1E-1	2.0E-2	1.9E-2	2.0E-1	2.0E-1
	FS	4.1E-4	5.5E-4	7.8E-4	9.2E-4	1.3E-2	1.5E-2	5.6E-2	5.8E-2	1.8E-2	2.1E-2	1.3E-1	1.3E-1
HGDP fit	PCA	1.0E-3	1.2E-3	1.3E-2	1.4E-2	2.3E-2	2.5E-2	1.2E-1	1.2E-1	3.4E-2	3.6E-2	2.2E-1	2.2E-1
	LFA	9.9E-4	1.1E-3	1.3E-2	1.2E-2	2.2E-2	2.4E-2	1.2E-1	1.2E-1	3.5E-2	3.7E-2	2.2E-1	2.2E-1
	ADX	1.6E-3	1.4E-3	2.3E-3	2.3E-3	2.6E-2	2.6E-2	5.6E-2	5.6E-2	3.6E-2	3.7E-2	1.0E-1	1.0E-1
	FS	1.4E-3	1.6E-3	3.1E-2	2.9E-2	2.6E-2	2.7E-2	1.4E-1	1.3E-1	3.6E-2	3.8E-2	2.2E-1	2.1E-1

Table S2: The top 50 SNPs most associated with structure in the HGDP data, identified by performing a logistic regression of SNP genotypes on the logistic factors. Shown are the SNP ID and location, deviance measure of differentiation, gene closest to the SNP, distance to gene (rounded to nearest 10bp), and the variant type (if none shown, then intergenic).

	rsid	chr	position	deviance	genesymbol	locusID	distance	variant type
1	rs1834640	15	48392165	1605.28	SLC24A5	283652	21000	
2	rs2250072	15	48384907	1313.82	SLC24A5	283652	28260	
3	rs12440301	15	48389924	1263.83	SLC24A5	283652	23240	
4	rs260690	2	109579738	1262.72	EDAR	10913	0	intron-variant
5	rs9837708	3	71487582	1189.48	FOXP1	27086	0	intron-variant
6	rs260714	2	109562495	1184.50	EDAR	10913	0	intron-variant
7	rs4918664	10	94921065	1178.40	XRCC6P1	387703	45340	
8	rs10882168	10	94929434	1160.99	XRCC6P1	387703	36970	
9	rs300153	2	17986417	1143.48	MSGN1	343930	11360	
10	rs9809818	3	71480566	1135.58	FOXP1	27086	0	intron-variant
11	rs6583859	10	94893473	1119.25	NIP7P1	389997	26290	
12	rs11187300	10	94920291	1114.22	XRCC6P1	387703	46120	
13	rs260698	2	109566759	1111.64	EDAR	10913	0	intron-variant
14	rs1834619	2	17901485	1111.40	SMC6	79677	0	intron-variant
15	rs11637235	15	48633153	1104.45	DUT	1854	0	intron-variant
16	rs4497887	2	125859777	1097.13	RNA5SP102	100873373	169180	
17	rs7091054	10	95018444	1085.45	RPL17P34	643863	25280	
18	rs7090105	10	75131545	1075.50	ANXA7	310	3640	
19	rs973787	4	38263893	1074.57	TBC1D1	23216	123090	
20	rs4279220	4	38254182	1070.43	TBC1D1	23216	113380	
21	rs7556886	2	17908130	1062.58	SMC6	79677	0	intron-variant
22	rs12473565	2	175163335	1056.31	LOC644158	644158	1390	
23	rs6500380	16	48375777	1051.10	LONP2	83752	0	intron-variant
24	rs2384319	2	26206255	1033.88	KIF3C	3797	810	upstream-variant-2KB
25	rs12220128	10	94975011	1023.79	XRCC6P1	387703	6090	
26	rs17034770	2	109616376	1019.03	EDAR	10913	10540	
27	rs3792006	2	26498222	998.96	HADHB	3032	0	intron-variant
28	rs4918924	10	94976956	994.79	XRCC6P1	387703	8030	
29	rs1984996	10	95008745	990.92	RPL17P34	643863	34980	
30	rs3751631	15	52534344	987.33	MYO5C	55930	0	reference,synonymous-codon
31	rs4578856	2	17853388	987.29	SMC6	79677	0	intron-variant
32	rs13397666	2	109544052	986.80	EDAR	10913	0	intron-variant
33	rs12619554	2	17352372	986.20	ZFYVE9P2	100420972	113180	
34	rs3736508	11	45975130	981.05	PHF21A	51317	0	missense,reference
35	rs12472075	2	177691130	973.02	RPL29P8	100131991	16650	
36	rs9522149	13	111827167	965.50	ARHGEF7	8874	0	intron-variant
37	rs2917454	10	78892415	964.40	KCNMA1	3778	0	intron-variant
38	rs10882183	10	94974083	961.04	XRCC6P1	387703	5160	
39	rs10079352	5	117494640	960.33	LOC100505811	100505811	123620	
40	rs10935320	3	139056584	958.33	MRPS22	56945	6270	
41	rs9571407	13	34886039	957.04	LINC00457	100874179	123540	
42	rs6542787	2	109556365	955.56	EDAR	10913	0	intron-variant
43	rs953035	1	36079508	954.67	PSMB2	5690	0	intron-variant
44	rs4657449	1	165465281	951.72	LOC400794	400794	0	intron-variant
45	rs9960403	18	13437993	949.43	LDLRAD4	753	0	intron-variant
46	rs203150	18	38037221	944.32	RPL17P45	100271414	312750	
47	rs2823882	21	17934419	942.05	LINC00478	388815	0	intron-variant
48	rs10886189	10	119753963	937.81	RAB11FIP2	22841	10460	
49	rs2441727	10	68224886	937.08	CTNNA3	29119	0	intron-variant
50	rs310644	20	62159504	931.90	PTK6	5753	260	downstream-variant-500B

Table S3: The top 50 SNPs most associated with structure in the TGP data, identified by performing a logistic regression of SNP genotypes on the logistic factors. Shown are the SNP ID and location, deviance measure of differentiation, gene closest to the SNP, distance to gene (rounded to nearest 10bp), and the variant type (if none shown, then intergenic).

	rsid	chr	position	deviance	genesymbol	locusID	distance	variant type
1	rs1426654	15	48426484	3129.76	SLC24A5	283652	0	missense,reference
2	rs3827760	2	109513601	2395.27	EDAR	10913	0	missense,reference
3	rs922452	2	109543883	2338.38	EDAR	10913	0	intron-variant
4	rs372985703	17	19172196	1975.16	EPN2	22905	0	intron-variant
5	rs4924987	17	19247075	1949.03	B9D1	27077	0	intron-variant,missense,reference
6	rs260687	2	109578855	1925.18	EDAR	10913	0	intron-variant
7	rs7209202	17	58532239	1890.67	APPBP2	10513	0	
8	rs7211872	17	58550725	1890.67	APPBP2	10513	0	
9	rs67929453	3	139109825	1890.57	LOC100507291	100507291	0	intron-variant,upstream-variant-2KB
10	rs260643	2	109539653	1850.71	EDAR	10913	0	intron-variant
11	rs260707	2	109574150	1838.37	EDAR	10913	0	intron-variant
12	rs1545071	18	67695505	1821.35	RTTN	25914	0	intron-variant
13	rs12729599	1	1323078	1812.91	CCNL2	81669	0	intron-variant
14	rs12347078	9	344508	1811.16	DOCK8	81704	0	intron-variant
15	rs12142199	1	1249187	1779.28	CPSF3L	54973	0	reference,synonymous-codon
16	rs12953952	18	67737927	1750.15	RTTN	25914	0	intron-variant
17	rs9467091	6	10651772	1746.75	GCNT6	644378	4270	
18	rs7165971	15	55921013	1736.83	PRTG	283659	0	intron-variant
19	rs6132532	20	2315543	1730.64	TGM3	7053	0	intron-variant
20	rs959071	17	19142226	1729.18	EPN2	22905	0	intron-variant
21	rs10962599	9	16795286	1726.24	BNC2	54796	0	intron-variant
22	rs967377	20	53222217	1724.93	DOK5	55816	0	intron-variant
23	rs4891381	18	67595449	1723.79	CD226	10666	0	intron-variant
24	rs377561427	15	63988357	1713.98	HERC1	8925	0	frameshift-variant,reference
25	rs73889254	22	46762214	1711.40	CELSR1	9620	0	intron-variant
26	rs4918664	10	94921065	1700.64	XRCC6P1	387703	45340	
27	rs2759281	1	204866365	1691.03	NFASC	23114	0	intron-variant
28	rs12065033	1	173579034	1682.54	ANKRD45	339416	0	utr-variant-3-prime
29	rs9796793	16	30495652	1681.28	ITGAL	3683	0	intron-variant
30	rs1240708	1	1335790	1675.48	LOC148413	148413	0	intron-variant,upstream-variant-2KB
31	rs2615876	10	117665860	1670.53	ATRNL1	26033	0	intron-variant
32	rs2823882	21	17934419	1669.32	LINC00478	388815	0	intron-variant
33	rs8097206	18	38024931	1663.29	RPL17P45	100271414	300460	
34	rs8071181	17	58508582	1662.44	C17orf64	124773	0	reference,synonymous-codon
35	rs1075389	15	64174177	1661.21	MIR422A	494334	10950	
36	rs6875659	5	175158653	1657.54	HRH2	3274	22410	
37	rs7171940	15	64170986	1654.01	MIR422A	494334	7760	
38	rs2148359	9	7385508	1652.16	RPL4P5	158345	91440	
39	rs7531501	1	234338303	1648.15	SLC35F3	148641	0	intron-variant
40	rs57742857	15	93567352	1645.21	CHD2	1106	0	intron-variant
41	rs931564	17	58631702	1636.86	LOC388406	388406	10200	
42	rs4738296	8	73857539	1632.70	LOC100288310	100288310	0	intron-variant
43	rs4402785	2	104766351	1631.33	LOC100287010	100287010	228950	
44	rs12988506	2	33162854	1630.14	LOC100271832	100271832	0	intron-variant
45	rs9410664	9	91196828	1625.48	NXNL2	158046	6120	
46	rs2041564	2	72453847	1623.91	EXOC6B	23233	0	intron-variant
47	rs6024103	20	54034601	1623.41	LOC101927796	101927796	2270	
48	rs6583859	10	94893473	1619.79	NIP7P1	389997	26290	
49	rs12913832	15	28365618	1611.23	HERC2	8924	0	intron-variant
50	rs632876	2	216572452	1610.26	LINC00607	646324	0	intron-variant
